# Effects of dietary crude protein levels in the concentrate supplement after grazing on rumen microbiota and metabolites by using metagenomics and metabolomics in Jersey-yak

**DOI:** 10.3389/fmicb.2023.1124917

**Published:** 2023-05-02

**Authors:** Rongfeng Dai, Xiaoming Ma, Renqing Dingkao, Chun Huang, Yongfu La, Xinyi Li, Xiaoyong Ma, Xiaoyun Wu, Min Chu, Xian Guo, Jie Pei, Ping Yan, Chunnian Liang

**Affiliations:** ^1^Key Laboratory of Yak Breeding Engineering Gansu Province, Lanzhou Institute of Husbandry and Pharmaceutical Science, Chinese Academy of Agricultural Sciences, Lanzhou, China; ^2^Key Laboratory of Animal Genetics and Breeding on Tibetan Plateau, Ministry of Agriculture and Rural Affairs, Lanzhou, China; ^3^Animal Husbandry Station, Gannan Tibetan Autonomous Prefecture, Gannan, Gansu, China

**Keywords:** protein level, compensatory feeding, rumen microbial, metagenome, metabolome, Jersey-Yak

## Abstract

**Introduction:**

The crude protein level in the diet will affect the fermentation parameters, microflora, and metabolites in the rumen of ruminants. It is of great significance to study the effect of crude protein levels in supplementary diet on microbial community and metabolites for improving animal growth performance. At present, the effects of crude protein level in supplementary diet on rumen fermentation parameters, microbial community, and metabolites of Jersey-Yak (JY) are still unclear.

**Methods:**

The purpose of this experiment was to study the appropriate crude protein level in the diet of JY. The rumen fermentation indexes (volatile fatty acids and pH) were determined by supplementary diets with crude protein levels of 15.16 and 17.90%, respectively, and the microbial community and metabolites of JYs were analyzed by non-target metabonomics and metagenome sequencing technology, and the changes of rumen fermentation parameters, microbial flora, and metabolites in the three groups and their interactions were studied.

**Results and Discussion:**

The crude protein level in the supplementary diet had significant effects on pH, valeric acid, and the ratio of acetic acid to propionic acid (*p* < 0.05). The protein level had no significant effect on the dominant microflora at the phylum level (*p* > 0.05), and all three groups were Bacteroides and Firmicutes. The results of metabolite analysis showed that the crude protein level of supplementary diet significantly affected the metabolic pathways such as Bile secretion and styrene degradation (*p* < 0.05), and there were different metabolites between the LP group and HP group, and these different metabolites were related to the dominant microbial to some extent. To sum up, in this experiment, the effects of crude protein level in supplementary diet on rumen microorganisms and metabolites of JY and their relationship were studied, which provided the theoretical basis for formulating a more scientific and reasonable supplementary diet in the future.

## 1. Introduction

Yak, as a unique animal species living on the Qinghai-Tibet Plateau, provides basic means of production and living for local herdsmen (Qiu et al., 2012). In order to improve the production performance of yak, artificial insemination was utilized to improve yak’s hybridization with high-quality frozen semen of Jersey and Angus cattle etc. However, the Qinghai-Tibet Plateau is in the grass-withering period from November to May of the following year. Climate change will affect the quality (mainly the content level of crude protein) and yield of forage grass. The nutrition in forage grass is not enough to meet the nutritional needs of yak and its hybrid offspring. Both yak and its hybrid offspring will suffer from a decline in productivity due to insufficient nutrition intake, and most importantly, they will reduce their weight, thus reducing the economic benefits of herders (Zhao and Zhou, 1999; Xue et al., 2005; Xu et al., 2017). Therefore, supplementary feeding during the dry season can appropriately reduce the loss of herders, and a reasonable protein level in the diet is one of the crucial measures to improve the growth performance and weight of animals.

As one of the main digestive organs for ruminants to degrade and digest the feed, the rumen is rich in microbial communities such as archaea, bacteria, fungi, and protozoa to ferment the feed (Zhao et al., 2022; Wang et al., 2022a; Xue et al., 2022a). The rich microbial community is closely related to the digestion and metabolism of the host feed, which can degrade and ferment organic substances such as plant fibers and polysaccharides into volatile fatty acids (VFAs), which are the principal energy sources of the host (Liu et al., 2021; Chen et al., 2022a; Xue et al., 2022a). Among them, the structure and function of the rumen microbial community are affected by many factors, such as variety, age, feeding mode, feeding level, etc. (Zhang et al., 2019; Li et al., 2020; Huang et al., 2021). 16S rRNA and metabonomics were used to study the bacterial composition in the rumen, and it was found that the nutritional level of diet could significantly change the relative abundance of dominant bacteria and metabolites in the rumen (Granja-Salcedo et al., 2016; Wang et al., 2020a, 2022b; Yi et al., 2022a). The level of protein in the diet will not only affect the composition of microorganisms and metabolites in the rumen but also affect the rumen fermentation indexes and serum biochemical indexes of the host (Wang et al., 2020b, 2022b).

Although the effects of dietary nutrient levels on rumen microbials and metabolites in ruminants have been extensively studied, most of them are limited to the level of rumen bacteria using techniques such as 16S rRNA. Moreover, due to the differences in digestive and metabolic characteristics and environmental adaptability between Jersey cattle, yak, and other cattle species, the effects on microbial communities and metabolites may also be different (Latham et al., 2018; Li et al., 2022; Pang et al., 2022a). With the deepening of microbial research, people gradually combine metagenomics and metabonomics to explore the effects of dietary nutrition level on host traits and rumen microbial community, (Xue et al., 2020, 2022a). Compared with 16S rRNA technology, the metagenomic can not only study the composition and diversity of bacteria in the host microbial but also conduct in-depth research on gene and function (Ghurye et al., 2016). However, when studying the effect of dietary nutrition level on Jersey-Yak (JY, hybrid offspring of female yaks by artificial insemination with high-quality frozen semen of Jersey), metagenomics and metabolomics have not been used for multi-omics research.

Therefore, in this study, in order to verify the influence of protein level in supplementary diet on rumen microbial community structure and metabolites of JYs, and the data of metagenomics, rumen metabolomics and serum metabolomics were integrated for joint analysis, the influence of protein level in supplementary diet on growth index and rumen fermentation parameters of JY was discussed. The correlation between different metabolites in serum and rumen and dominant microflora in rumen was analyzed, and the microflora and metabolism were discussed. Provide more scientific guidance for fattening Jersey, and help herders acquired more economic benefits.

## 2. Materials and methods

The animal experiments involved in this experiment were approved by the Lanzhou Institute of Husbandry and Pharmaceutical Sciences of the Chinese Academy of Agricultural Sciences (CAAS; approval number: 1610322020018). All sampling procedures are strictly in accordance with the Guidelines for Ethical Treatment of Experimental Animals in China.

### 2.1. Animal and experimental design

The experiment was conducted in Xiahe County, Gannan Tibetan Autonomous Prefecture. In this experiment, 18 healthy 6-month-old male JYs were selected, and their weight before the preliminary experiment was 62.20 ± 2.64 kg. According to the weighing data before the pre-experiment, 18 JYs were randomly divided into three treatment groups by R software (Version 4.1.2). Feeding management is as follows: each group grazes at 9:00 a.m. and 6:00 p.m. every day, all grazing in the same pasture. After grazing every day, each group was raised in a separate column, and then the treatments of the three groups were: (1) No supplementary feeding, grazing (as control group, Control); (2) Supplementing low-protein diet (protein content: 15.16%; LP); (3) Supplementing high-protein diet (protein content: 17.90%; HP). See Supplementary material S1 for the composition and content of supplementary diet. Among them, the supplementary feeding amount is adjusted according to the monthly weighing data of Juangu cattle, and the monthly supplementary feeding amount is calculated according to 1.2% of the weight of JY in the HP and LP group.

This experiment includes two stages: pre-experiment (15 days) and formal experiment (120 days). The purpose of the experiment is to make the experimental animals adapt to the supplementary diet in advance. After the end of the pre-experiment and the beginning of the formal experiment, the weight of JY was measured regularly every month.

### 2.2. Sample collection

After the formal experiment, JYs in each group fasted for 1 day. The blood of each JY was collected by jugular vein puncture with a blood collection tube (5 ml) without additives, then centrifuged at 4500 × *g* for 15 min with a portable medical centrifuge to separate the serum. The separated serum samples were stored in foam boxes with ice bags, transported back to the laboratory and stored in a refrigerator at −20°C for biochemical index analysis. After blood samples were collected, 150 ml rumen fluid samples of each JY were collected by oral stomach tube. After filtration, it was distributed into a 50 ml centrifuge tube, then immediately placed in a liquid nitrogen tank. After the sample was sent to the laboratory, it was stored in a refrigerator at −80°C for subsequent metagenome sequencing analysis and determination of VFAs.

### 2.3. Determination of rumen fermentation parameters

After the rumen fluid was collected, it was filtered with four layers of gauze, and then the pH of the filtered rumen fluid was measured with a portable pH meter. Each sample was repeated three times. Rumen liquid transported back to the laboratory was thawed on ice, and centrifuged for 15 min at 4°C and 10000 × *g* by low-temperature freezing centrifuge. The supernatant was transferred to a solution containing an internal standard (2-Ethylbutyric acid, 2 EB) and a concentration of 25% metaphosphoric acid. Then the concentration of VFAs was determined by gas chromatography using the method introduced by Erwin et al. (1961).

### 2.4. Determination and analysis of serum and rumen fluid non-target metabolome

The rumen fluid sample (100 μl) was sucked into a 1.5 ml centrifuge tube, and 400 ml of extractive solution (acetonitrile: methanol = 1:1) was added, respectively, for low-temperature ultrasonic extraction (5°C, 40 kHz); Standing at < 20°C for 30 min; Centrifuge at 4°C, 13000 × *g* for 15 min, remove the supernatant, add 100 μl complex solution (acetonitrile: water = 1:1) for redissolving, and perform low-temperature ultrasonic extraction; Centrifuge at 4°C and 13000 × *g* for 5 min, and suck the supernatant into the sample bottle for computer analysis.

The obtained raw data were filtered by the software Progenesis QI (Waters Corporation, Milford, United States) for baseline, peak identification, integration, and retention time correction, etc., and the data matrix of retention time, mass-to-charge ratio, and peak intensity was obtained. Then, the data matrix was compared with HMDB^1^, and the information on metabolites was obtained. Data pre-processing: pre-processing the matrix data, mainly including missing value filtering, filling in vacant values, data normalization, quality control sample verification, and data conversion. During the verification of quality control samples, the data with relative standard deviation (RSD) > 30% are mainly deleted and not used for subsequent analysis.

According to the expression of metabolites in different groups of samples, partial least squares discriminant analysis (PLS-DA) was performed on the samples by using the “Vegan” package of R software (Version 1.6.2) to judge the degree of separation between the samples. According to the variable importance in the projection (VIP) value obtained by PLS-DA analysis, the Kruskal–Wallis H test was used to screen the different metabolites among the three groups. The difference metabolites between the LP group and HP were judged by the Wilcoxon rank sum test, and they were all realized by the “stats” package of R software, in which *p* < 0.05 was regarded as statistically significant. The annotation of metabolites was obtained by using the Kyoto Encyclopedia of Genes and Genomes (KEGG) and HMDB, and then the “scipy” in Python (Version 1.0.0) was used to analyze the metabolic pathway and enrichment of target metabolites.

### 2.5. DNA extraction, metagenome sequencing, and data processing

Total genomic DNA from rumen fluid was extracted by repeated bead beating plus column (RBB + C) method introduced by Yu and Morrison (2004). Then the quality of the extracted DNA was detected by 1% agarose gel electrophoresis, and the DNA concentration was measured by ultraviolet spectrophotometer. The sequencing of macro genome was completed in Shanghai Meiji Biomedical Technology Co., Ltd. Covaris M220 was used for fragmentation (about 400 bp in length), PE library was constructed according to the instructions of Next Flex Rapid DNA-SeqKit (Bioo Scientific, United States), Nova Seq Reagent Kits/His EQ X Reagent Kits were used for bridge PCR and Illumina Novaseq6000 instrument was used for sequencing. All the raw data were submitted to the NCBI Sequence Read Archive (SRA) database (Accession number: PRJNA904725).

Make statistics and quality control on the original reads obtained by sequencing, and obtain high-quality reads to ensure the accuracy of subsequent analysis. It mainly uses FASTP (Version 0.23.0, https://github.com/OpenGene/fastp) software to cut the adapter sequences at the 3′ and 5′ ends of the sequences and remove the low-quality reads (the length is less than 50 bp, the average mass value is less than 20, and it contains N bases). After quality control, the high-quality data were assembled by MEGAHIT (Version 1.1.2; contig ≥300 bp), and the open reading frame (ORF) in the splicing result was predicted by Prodigal (Version 2.6.3; the sequence length was > 100 bp), and it was translated into the amino acid sequence (Hyatt et al., 2010; Li et al., 2015). Using CD-HIT software (Version 4.6.1) to cluster the predicted gene sequences (identity > 90%, coverage > 90%) to obtain gene sets (Fu et al., 2012). Using SOAPaligal software (Version: 2.21), the gene abundance (RPKM) was calculated by comparing each sample with the gene set by formula (Li et al., 2009; Lawson et al., 2017).

Use Diamond software (Version 2.0.13) to compare the gene set with the RefSeq database (parameter: *E*-value ≤ 1*e*–5) to make a taxonomic annotation on rumen microorganisms at the phylogenetic level, and compare with KEGG to obtain the corresponding functional annotation information of the samples (Buchfink et al., 2015, 2021). The annotation of carbohydrate-active enzyme (CAZyme) genes was obtained by using HMMER (Version 3.1b2) software against carbohydrate-active enzyme database (hmmscan; *E*-value ≤ 1*e*–05).

According to the gene abundance calculated by the RPKM method, the microbial composition at the phylum and genus level was analyzed and mapped by using the vegan package in R (Version: 4.1.2). Column diagram was used to visualize the microbial composition of the three treatment groups at phylum and genus level. Among the three treatment groups, microbials with significant differences between the LP group and the HP group were subjected to the Kruskal–Wallis H test by using the stats package of R software (Version: 4.1.2).

### 2.6. Correlation analysis

According to VIP > 1, *p* < 0.05, the correlation between different metabolites selected from serum and rumen fluid and rumen fermentation parameters (acetic acid, propionic acid, *iso*-butyric acid, butyric acid, *iso*-valeric acid, pentanoic acid, and hexyl-propyl ratio) was analyzed. SciPy of Python (Version 1.0.0) was used to analyze the Spearman correlation between different metabolites and rumen fermentation parameters based on the Bray_Curtis distance, and the cluster heat map was used for visualization.

Metabolites in serum and rumen with VIP > 1, *p* < 0.05 were interactively analyzed with the top 50 dominant microflora in phylum and genus taxonomy. Based on Bray_Curtis distance, the Spearman correlation between differential metabolites and dominant microbial phylum and dominant microbial genus was analyzed by using “scipy” of Python (Version 1.0.0), and these correlations were visualized by cluster heat map.

### 2.7. Statistical analysis

SAS software (Version 9.4) was used to analyze the monthly weight data and weight changes of each group of JY designed by random block with one-way analysis of variance (ANOVA). The statistical results were expressed by mean and standard deviation. Then use GraphPad (Version 9.0) for visualization. Blood biochemical indexes and VFAs were tested by SPSS software (Version 26) for normality and homogeneity of variance, and ANOVA was used to analyze the differences among the three groups, among which *p* < 0.05 was regarded as statistically significant. Finally, the results were expressed by the standard deviation (SD) of the mean value.

## 3. Results

### 3.1. Changes in growth index

**Figure 1:**
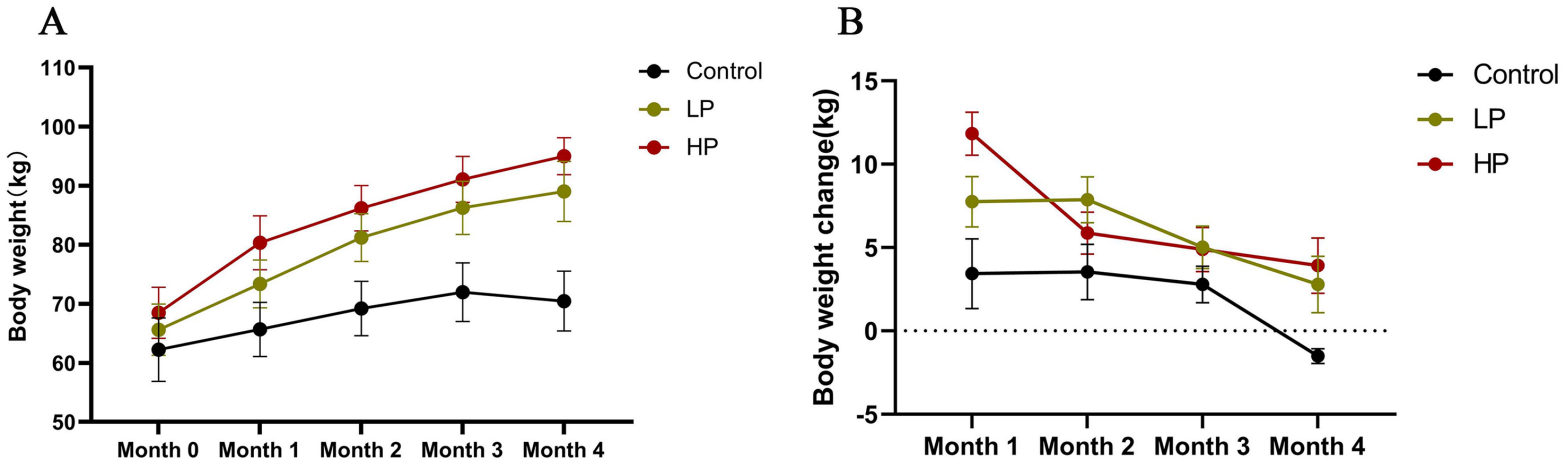
Effects of dietary protein level on body weight **(A)** and body weight change **(B)**. The mean value and standard deviation (SD) of each group were used to represent. Control: control group, no supplementary feeding; LP: low protein group, supplementary feeding with low protein diet; HP: high protein group, supplementary feeding with high protein diet.

The effects of different protein levels of supplementary diet on the body weight and monthly body weight change of JY during the formal trial period are shown in Figure 1. According to SAS, ANOVA (Supplementary material S2) was carried out on the body weight and weight changes of the three groups. The results showed that there were significant differences in the body weight each month among the three groups (*p* < 0.05), and the body weight of the HP group was higher than that of the LP group and Control group. In terms of the monthly weight gain rate, JY of the LP group and HP group with supplementary feeding were in a state of weight gain in the formal experiment, but the weight of the control group without supplementary feeding showed a significant downward trend in the last month of the formal experiment.

### 3.2. Changes of rumen fermentation parameters

The effects of three treatments on rumen fermentation parameters of JYs are shown in Table 1. The valeric acid content and pH of the LP and HP groups were significantly higher than those of the Control group (*p* < 0.05). The isopropyl ratio of the Control group was significantly higher than the other two groups (*p* < 0.05). There was no significant difference in the content of acetic acid, valeric acid, *iso*-butyric acid, butyric acid, and *iso*-valeric acid among the three groups (*p* > 0.05).

**Table 1:** Effects of supplemental diets with different protein levels on rumen fermentation parameters of Jersey-Yak.

Item	Treatment group (Mean ± SD)			*p*-Value
	Control	LP	HP	
Acetate	33.02 ± 5.52	35.75 ± 10.33	34.40 ± 3.82	0.628
Propionate	6.68 ± 1.18	8.40 ± 2.62	7.91 ± 1.91	0.112
*Iso*-butyrate	0.31 ± 0.04	0.37 ± 0.08	0.34 ± 0.08	0.105
Butyrate	4.16 ± 1.08	5.84 ± 2.79	4.96 ± 1.16	0.086
*Iso*-valerate	0.32 ± 0.07	0.41 ± 0.10	0.37 ± 0.13	0.095
Valerate	0.26 ± 0.05^b^	0.47 ± 0.24^a^	0.36 ± 0.16^ab^	0.021
Acetate: Propionate	4.96 ± 0.44^a^	4.35 ± 0.63^b^	4.49 ± 0.67^b^	0.040
pH	6.57 ± 0.17^B^	6.92 ± 0.18^A^	7.02 ± 0.16^A^	0.006

The value of each treatment group is expressed by the Mean ± standard deviation (SD). In the same line, superscript with different lowercase letters shows significant differences among groups, while uppercase letters show significant differences among groups.

### 3.3. Statistics of metagenomic data

A total of 864,673,934 raw reads were obtained from 18 samples in three treatment groups, with an average of 48,037,441 ± 4,449,071 raw reads per sample. After quality control, 851,239,334 clean reads were retained, with an average of 47,291,074.11 ± 4,397 clean reads per sample (Supplementary material S3). A total of 11,800,805 contigs were generated by *de novo* assembly of the retained data, with an average of 655,600.28 ± 81,518.10 contigs per sample, of which the length of N50 per sample was 772.00 ± 99.47 bp. Gene prediction showed that there were 15,548,998.00 open reading frame ORFs in all samples (Supplementary material S3). Bacteria accounted for 96.67%, eukaryotes accounted for 1.35%, archaea accounted for 1.07%, and viruses accounted for 0.59% (Supplementary material: S4).

### 3.4. Microbial maps of three treatment groups

**Figure 2:**
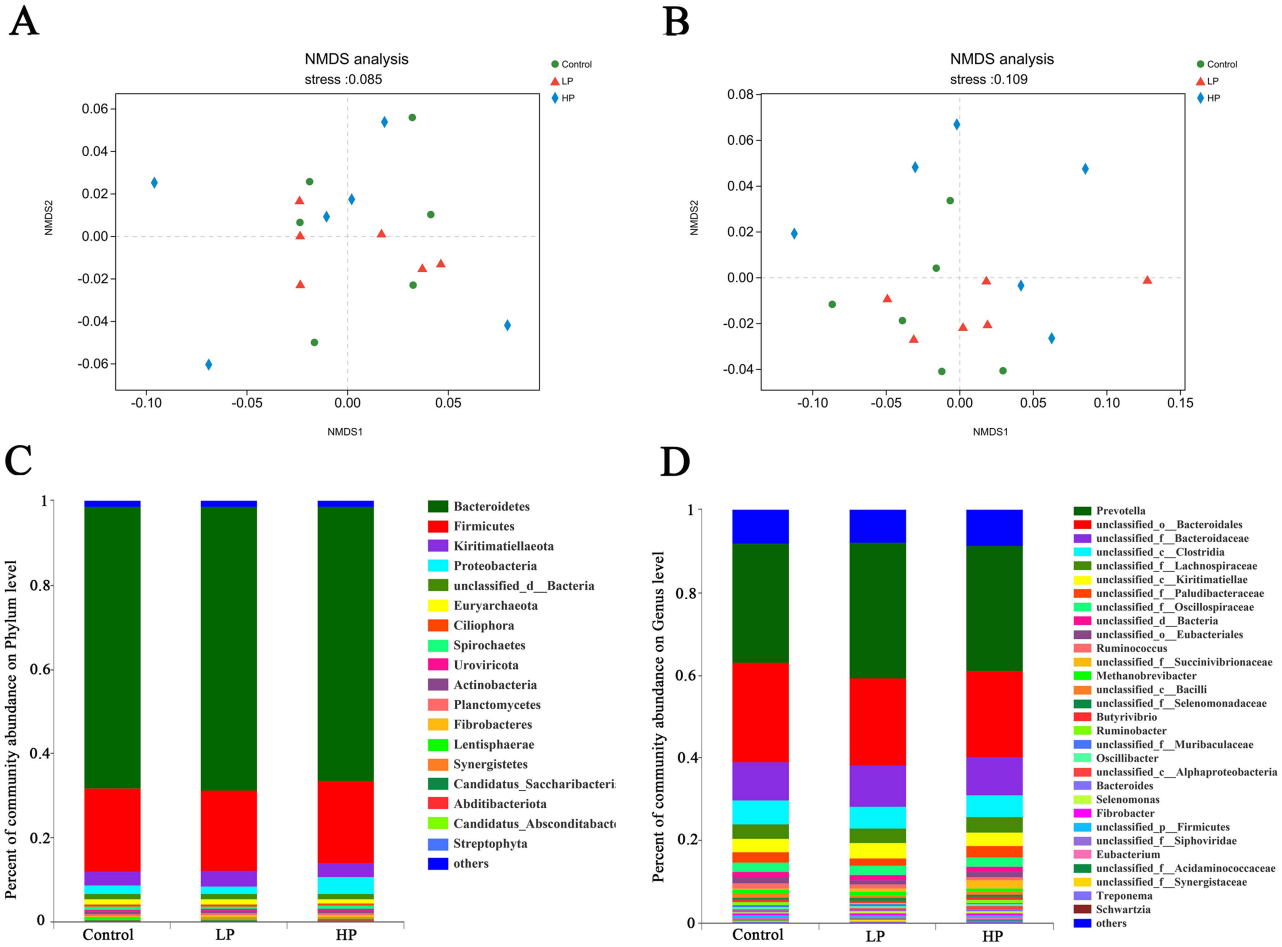
Non-metric multi-dimensional analysis (NMDS) of metagenome sequencing and microbial composition at genus and phylum level in three treatment groups. NMDS of metagenome sequencing of all samples at phylum **(A)** and genus **(B)** level. Microbial composition profiles of three treatment groups at phylum **(C)** and genus **(D)** levels.

The microbial composition of 18 samples (at Phylum and Genus levels) was analyzed by non-metric multidimensional scale analysis (NMDS), which was analyzed and plotted by the vegan package of R software (Figures 2A,B). The results showed that the three treatment groups had a good clustering effect, which could be followed up. According to the visualization of the microbial composition of the three treatment groups at the phylum and genus level (Figures 2C,D), the results showed that at the phylum level, the dominant phylum of the three treatment groups was *Bacteroidetes*, *Firmicutes*, *Kiritimatiellaeota*, and *Proteobacteria*, etc. At the genus level, the dominant microbial genera in the three treatment groups mainly include *Prevotella*, *unclassified_o_bacteroidales*, *unclassified_f_lachnospiraceae*, *unclassified_c_kiritimatiellae*, *unclassified_f_paludibateraceae*, *Ruminococcus*, and *Methanobrevibacter*.

**Figure 3:**
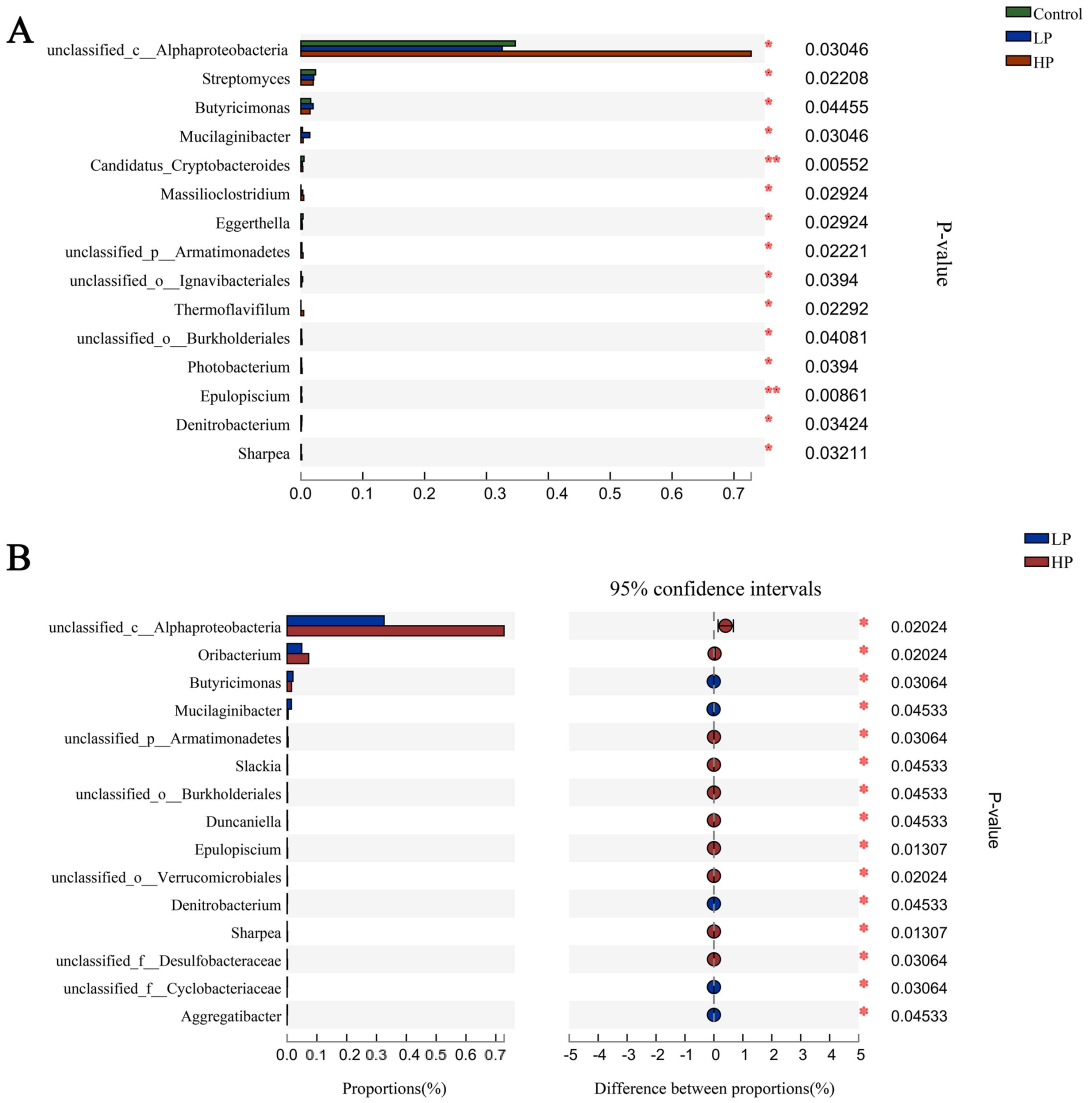
Composition differences of microbials in three treatment groups and LP_HP groups at genus level. **(A)** The difference of microbial composition in three treatment groups at genus level. **(B)** Difference of microbial composition between LP and HP groups at genus level. “*” indicates that metabolites are significantly different among groups. “**” indicates that there is a significant difference in metabolites between groups.

In this study, based on the difference matrix of Bray_Curtis, the difference of microbial composition in three treatment groups was estimated (Figure 3A). The content of *unclassified_c_alphabetobacter* in HP group was significantly higher than that in the other two groups. Besides, the contents of *Streptomyces*, *Butyricimonas*, *Mucilaginibacter*, *Candidatus_Cryptobacteroides*, *Massilioclostridium*, and other microbials were also significantly different among the three treatment groups. In the comparison of microbials between LP and HP groups (Figure 3B), the contents of *Oribacterium*, *Slackia*, *unclassified_o_burkholderiales*, *Duncaniella*, and *epulopsis* in HP group were significantly higher than those in LP group.

### 3.5. Enzyme map of carbohydrate activity in three treatment groups

**Figure 4:**
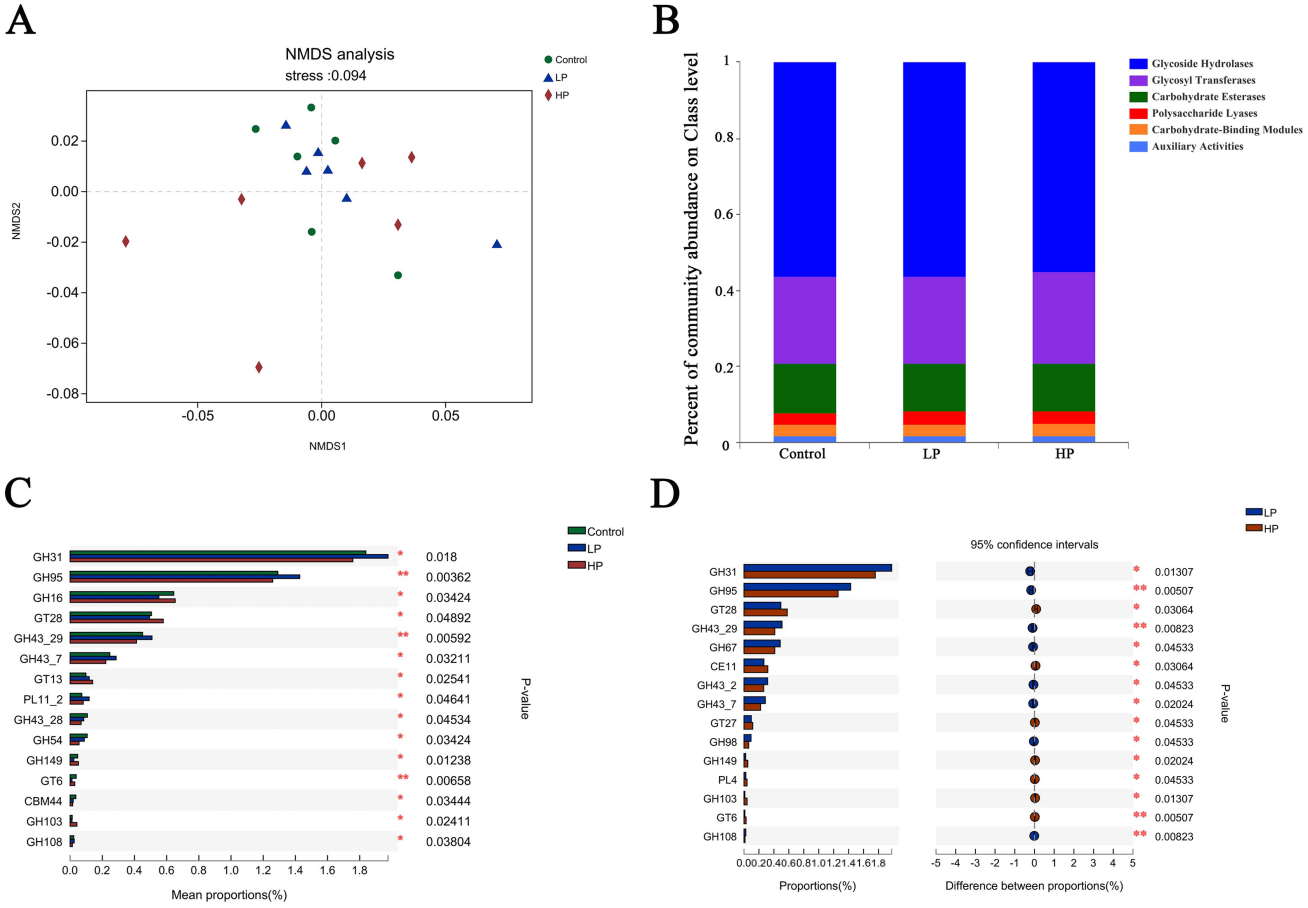
Non-metric multi-dimensional analysis of carbohydrate-active enzymes **(A)** and composition of three treatment groups **(B)**. There are significant differences in CAZymes between the three treatment groups **(C)** and LP_HP two treatment groups **(D)**.

The function of microbial is partly determined by genes encoding carbohydrate-active enzymes (CAZymes). The genes encoding CAZymes were analyzed by NMDS (Figure 4A), and the results showed that the genes encoding CAZymes in three treatment groups had a good clustering effect. A total of 527 CAZyme genes were identified (Figure 4B), including 260 Glycoside Hydrolases (GHs), 87 Glycosyl Transferases (GTs), and 77 Polysaccharide Lyases (PLs). There are 6 Carbohydrate Esterases (CEs), 70 Carbohydrate-Binding Modules (CBMs), and 17 and auxiliary oxidoreductases (AAs). Among them, the genes encoding glycosidase GHs and GTs are the most.

The differences of CAZyme coding genes among three treatment groups were compared (Figure 4C). The results showed that the relative abundance of GT28, GT13, GH149, and GH103 in HP group was significantly higher than that in other two groups, and the relative abundance of GH31, GH95, GH43_29, and PL11_2 in LP group was significantly higher than that in other two groups. Compared with HP group (Figure 4D), the results showed that the contents of GH31, GH95, GH43_29, GH67, and GH98 in LP group are significantly higher than those in HP group, and the relative abundance of GT28, CE11, GT27, and GH149 in HP group is significantly higher than that in LP group.

### 3.6. Serum and rumen fluid metabolomics

**Figure 5:**
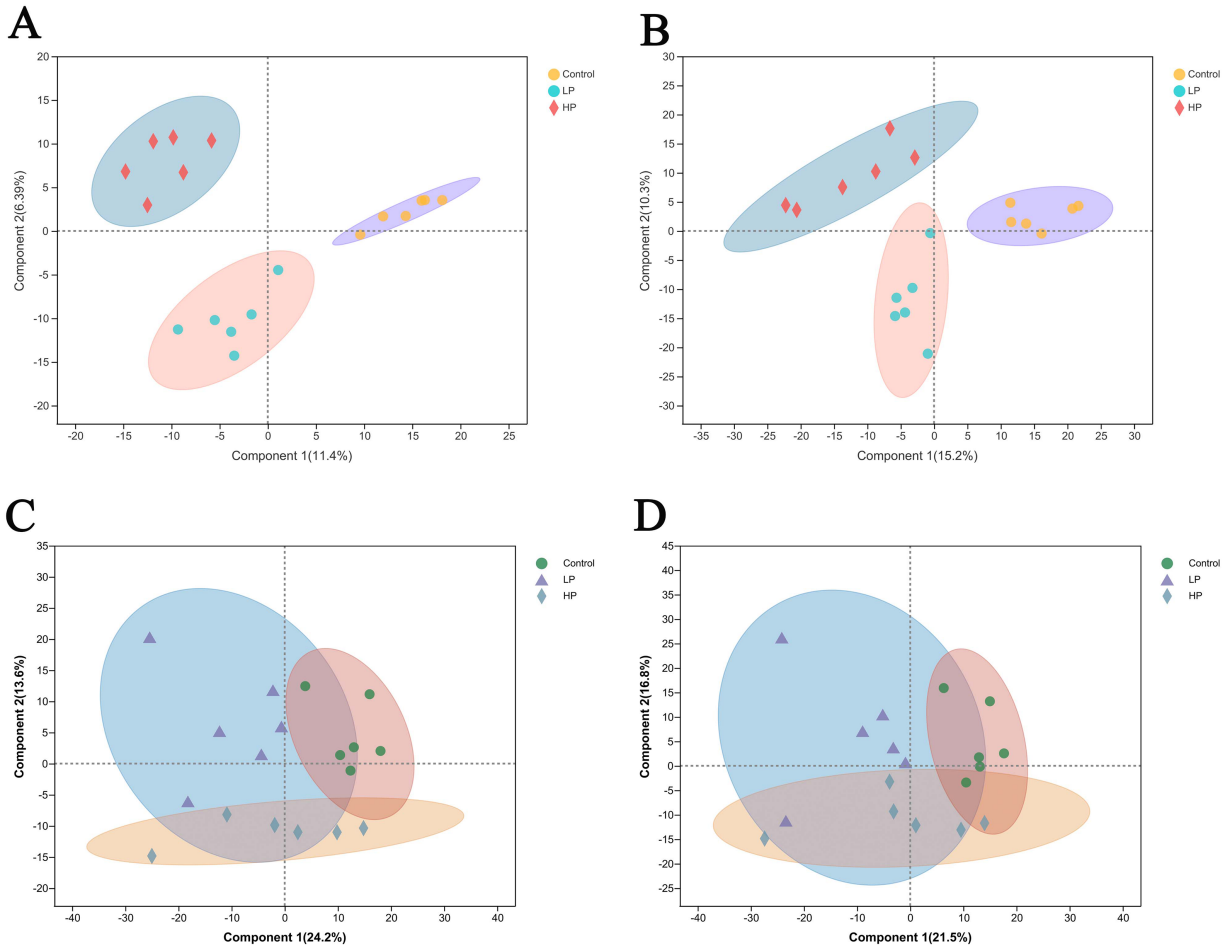
Partial least squares discriminant analysis (PLS-DA) scores of serum metabolism group and rumen metabolism group. PLS-DA analysis of serum metabolites in three treatment groups under cation mode **(A)** and anion mode **(B)**. PLS-DA analysis of rumen metabolites in three treatment groups under cation mode **(C)** and anion mode **(D)**.

Metabolites were detected in the serum of three treatment groups, and 884 metabolites were obtained. According to the PLS-DA scores of metabolites in the serum of three treatment groups in cation mode and anion mode (Figures 5A,B), the results show that the clustering effect of samples in the group is good, which can be used for subsequent analysis. Among them, after the Kruskal–Wallis H test, 125 metabolites were screened out among the three treatment groups, and there were significant differences among 48 metabolites between LP and HP groups after Wilcox’test (*p <* 0.05 VIP > 1).

**Figure 6:**
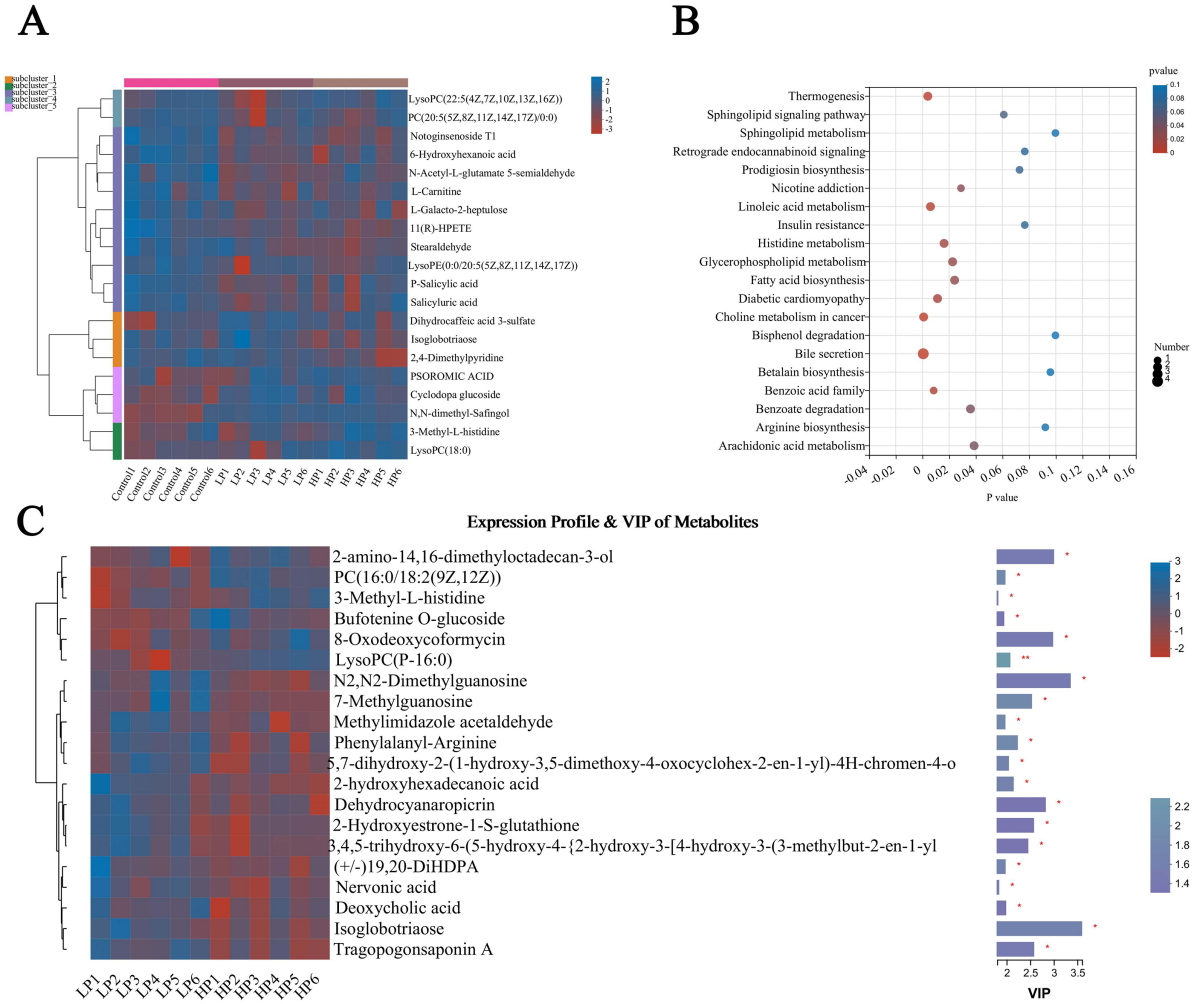
Differential metabolites and metabolic pathways in serum of three treatment groups. Differential metabolites in serum of three treatment groups **(A)** were visualized by cluster thermogram. Bubble chart was used to show the different metabolic pathways in the serum of the three treatment groups **(B)**. Differential metabolites in serum of LP and HP **(C)** were visualized by thermogram and VIP value. The tree diagram of metabolites cluster on the left side shows that each column shows a sample, the lower side shows the name of the sample, and each row shows a metabolite. Bar chart showing VIP value on the right. “*” indicates that metabolites are significantly different among groups. “**” indicates that there is a significant difference in metabolites between groups. The same below.

The products with the top 20 abundance were selected and represented by cluster heat map (HCA; Figure 6A). The results showed that the differential metabolites could be clustered into five clusters. The first cluster mainly included two metabolites, LysoPC [22:5(4Z, 7Z, 10Z, 13Z, 16Z)] and PC [20:5(5Z, 8Z, 11Z, 14Z, 17Z)/0:0]. The second cluster mainly includes 10 metabolites including Notoginsenoside T1, 6-Hydroxyhexanoic acid, N-acetyl-L-glutamate5-semi aldehyde, and L-Carnitine. The third cluster mainly includes three metabolites of Dihydrocaffeic acid 3-sulfate, Isoglobotriaose, and 2,4-Dimethylpyridine, the fourth cluster mainly includes PSOROMIC ACID, Cyclodopa glucoside, and N, N-dimethyl-Safingol, and the fifth cluster mainly includes 3-Methyl-L-histidine and lysopc. The different metabolic pathways among the three treatment groups (Figure 6B) mainly include Sphingolipid metabolism, Bile secretion, Linoleic acid metabolism, Histidine metabolism, and so on. According to the VI*p* value and *P* value obtained by PLS-DA analysis, the differential metabolites between LP and HP groups were screened (Figure 6C). The results showed that there were mainly 2-amino-14,16-dimethyloctodecan-3-ol, Isoglobotriaos, Tragopogonsaponin A, and 2-hydroxyhexadecanoic acid between the two treatment groups.

Metabolites in rumen fluid of three treatment groups were detected, among which 1,667 metabolites were identified. According to the PLS-DA scores of metabolites in the rumen of three treatment groups in cation mode and anion mode (Figures 5C,D), the results showed that the clustering effect of samples in the group is good, which can be used for subsequent analysis. As with the analysis method of metabolites in serum, 204 metabolites were significantly different among the three treatment groups, and 45 metabolites were significantly different between LP and HP groups.

**Figure 7:**
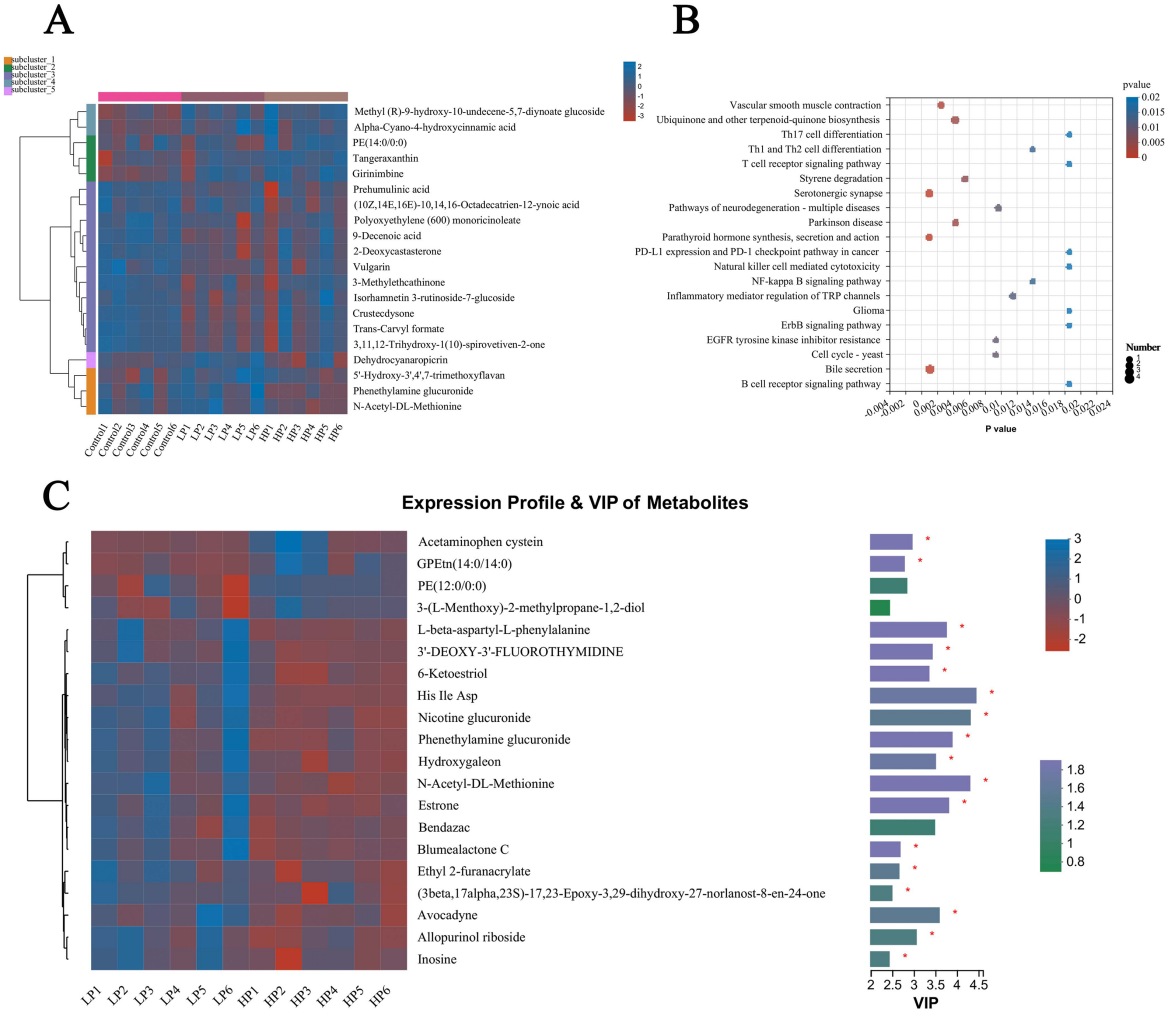
Differential metabolites and metabolic pathways in rumen fluid of three treatment groups. Differential metabolites in rumen fluid of three treatment groups **(A)** were visualized by cluster thermogram. Bubble diagram was used to show the different metabolic pathways in rumen fluid of three treatment groups **(B)**. Differential metabolites in rumen fluid of LP group and HP group **(C)** were visualized by heat map and VIP value. The tree diagram of metabolites cluster on the left side shows that each column shows a sample, the lower side shows the name of the sample, and each row shows a metabolite. Bar chart showing VIP value on the right. “*” indicates that metabolites are significantly different among groups. “**” indicates that there is a significant difference in metabolites between groups.

The top 20 metabolites in abundance were selected and visualized by cluster thermogram (Figure 7A). The results show that the different metabolites in rumen fluid can be clustered into five clusters, the first cluster contains methyl (r)-9-hydroxy-10-undecene-5, 7-diynoate glucoside and Alpha-Cyano-4-hydroxycinnamic acid are two metabolites, and the second cluster contains three differential metabolites of PE (14.0/0:0), Tangeraxanthin and Girinimbine. The third cluster contains 11 metabolites including Prehumulinic acid, 9-Decenoic acid, and 2-deoxycastastrone, the fourth cluster contains a metabolite of Dehydrocyanaropicrin, and the fifth cluster contains 5′-Hydroxy-3′,4′, 7-Trimethoxylavan, Phenethylamine glucuronide, and N-Acetyl-DL-Methionine, three metabolites. According to the analysis of the different metabolic pathways among the three treatment groups (Figure 7B), the results showed that there were mainly different metabolic pathways such as Th17 cell differentiation, Styrene degradation, and Bile secretion. According to the VI*p* value and *P* value obtained by PLS-DA analysis, the different metabolites in the rumen fluid of LP and HP groups were screened (Figure 7C). The results showed that there were significant differences in metabolites such as Acetaminophen cysteine, Nicotine glucuronide, Phenethylamine glucuronide, Hydroxygaleon, and N-Acetyl-DL-Methionine between the two groups (LP and HP).

### 3.7. Relationship between different metabolites in serum and rumen fluid and rumen fermentation parameters

**Figure 8:**
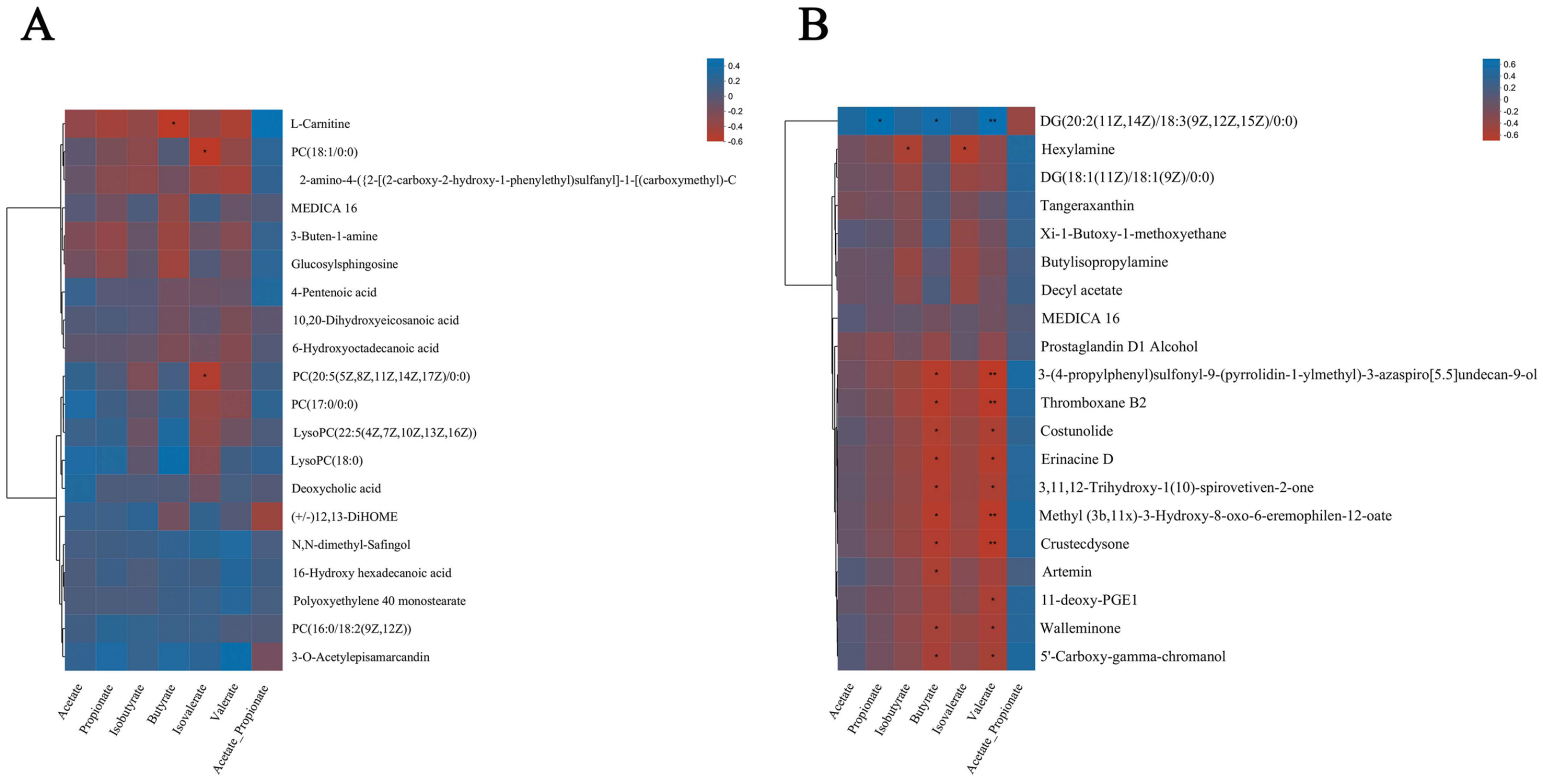
Spearman relationship between different metabolites in serum and rumen fluid and VFAs. Spearman correlation between different metabolites screened from serum **(A)** and rumen fluid **(B)** and VFAs in three treatment groups.

To determine the difference metabolites in serum and rumen fluid and rumen fermentation parameters (acetate, propionate, *iso*-butyrate, butyrate, *iso*-valerate, valerate, and acetic/propionic ratio), Spearman rank correlation analysis was carried out on the difference metabolites, and these parameters. According to the correlation analysis of differential metabolites in the serum and VFA parameters (Figure 8A), results showed that Acetate, Propionate, Butyrate, and L-Carnitine, PC (18:1/0:0), 2-amino-4-({2-[(2-carbon-2-hydroxy-1-phenyl ethyl) sulfinyl]-1-(carbo) Metabolites such as 3-Buten-1-amine and Glucosylsphingosine were negatively correlated with N,N-dimethyl-Safingol, 16-Hydroxy hexadecanoic acid, Polyoxyethylene 40 monostearate and PC [16:0/18:2(9Z, 2z)] and other metabolites showed positive correlation, among which Butyrate and L-Carnitine had extremely significant negative correlation (*p* < 0.05), Isovalerate and PC (18:1/0:0) and PC [20:5(5Z,8Z,11Z,14Z,17Z)/0:0]. Isopropyl ratio is positively correlated with most of the differential metabolites.

In the analysis of the correlation between different metabolites in rumen fluid and VFAs (Figure 8B), the results show that acetate, propionate, *iso*-butyrate, butyrate, *iso*-valerate, and valerate are negatively correlated with most of the different metabolites, in particular, butyrate and valerate are negatively correlated with metabolites such as Thromboxane B2, Costunolide and Erinacine D (*p* < 0.05), but the indexes of propionate, butyrate and valerate are correlated with metabolites DG [20: 2 (11z, 14z)/18: 3 (9z, 12z, 15z]/. *Iso*-propyl ratio showed a negative correlation with DG [20: 2 (11Z, 14Z)/18: 3 (9Z, 12Z, 15Z)/0: 0] and other metabolites, but showed a positive correlation with most metabolites.

### 3.8. Relationship between rumen dominant microflora and rumen fermentation parameters and metabolites

**Figure 9:**
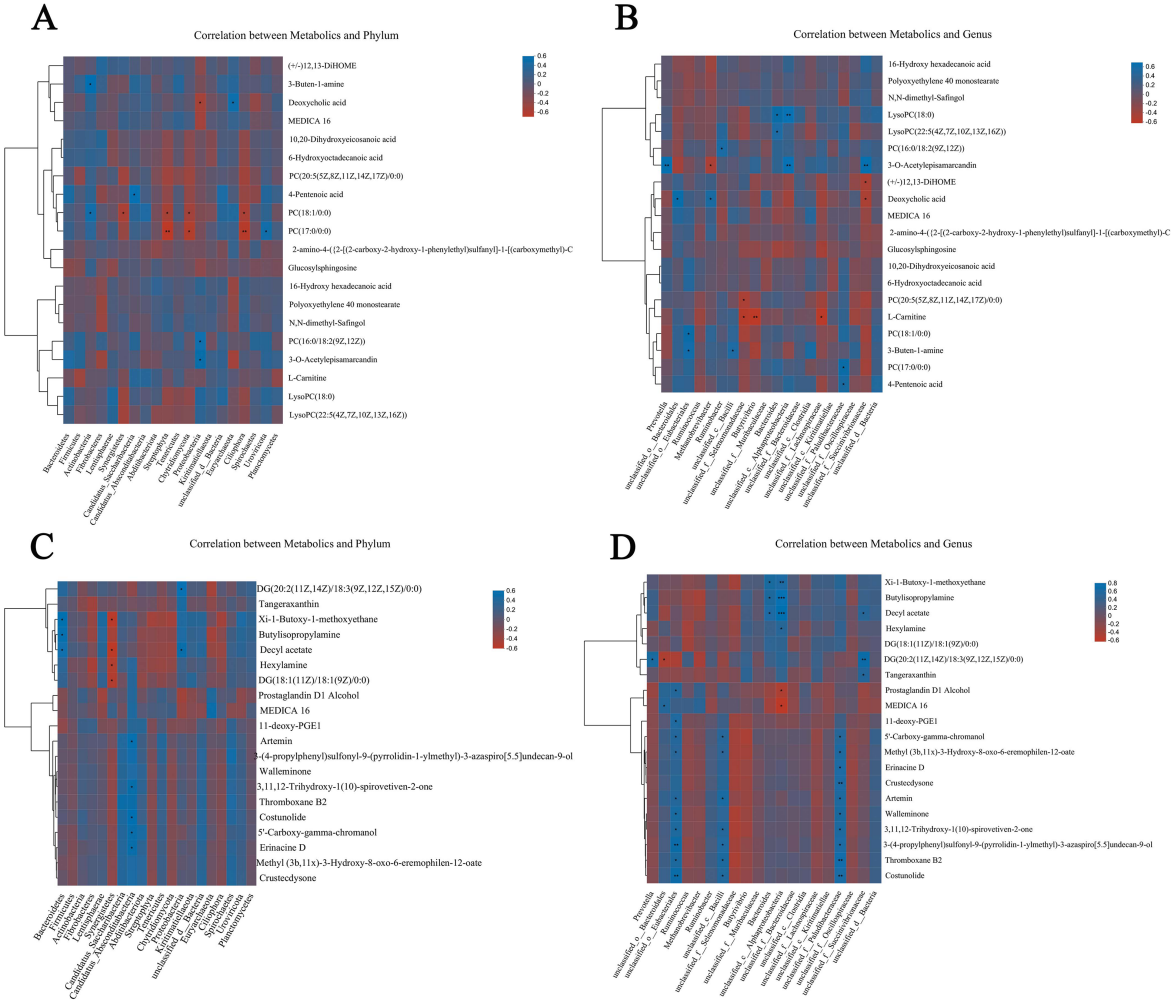
Correlation among rumen metagenome, serum and rumen metabolomics. Spearman level correlation between rumen microflora and metabolites. Correlation analysis between dominant microbials and serum differential metabolites at taxonomic level of phylum **(A)** and genus **(B)**. Correlation between dominant microflora at taxonomic level of phylum **(C)** and genus **(D)** and differential metabolites in rumen fluid.

To determine the potential interaction between rumen microflora and host metabolism, the dominant microflora with the top 20 phylum and genus levels were selected to correlate with Spearman rank between different metabolites in serum and rumen fluid, respectively. According to the correlation analysis between serum differential metabolites and dominant microflora (Figures 9A,B), the results show that at the phylum level, *Bacteroidetes*, *Firmicutes*, *Actinobacteria*, and *Fibrobacteres* are negatively correlated with 10,20-Dihydroxyeicosanoic acid and 6-hydroxytadecanoic acid, while other dominant microflora are positively correlated with these two metabolites. *Actinobacteria* showed an obvious positive correlation with metabolites such as 3-Buten-1-amine, Deoxycholic acid, MEDICA 16, 10,20-Dihydroxyeicosanoic acid, and 6-hydroxytadecanoic acid, There was a significant negative correlation with metabolites such as 16-Hydroxy hexadecanoic acid, Polyoxyethylene 40 monostearate and N,N-dimethyl-Safingol, and there was a significant positive correlation with metabolites such as 3-Buten-1-amine and PC (18:1/0:0; *p* < 0.05). Metabolites such as PC (18:1/0:0) and PC (17:0/0:0) have significant negative correlations with dominant phylum such as Streptophyta and Chytridiomycota (*p* < 0.05). At the genus level, *Prevotella* has a negative correlation with most of the differential metabolites, and a very significant positive correlation with 3-o 3-O-Acetylepisamarcandin (*p* < 0.01). The dominant microbial phylum *Ruminococcus* and *Methanobrevibacter* were positively correlated with the metabolites of 16-Hydroxy hexadecanoic acid and Polyoxyethylene 40 monostearate, among which *Methanobrevibacter* was positively correlated with Deoxycholic acid (*p* < 0.05).

According to the correlation analysis between the differential metabolites in rumen fluid and the dominant microflora (Figures 9C,D), the results show that: at the phylum level, *Bacteroidetes* showed an obvious negative correlation with most metabolites except Tangeraxan, Xi-1-Butoxy-1-methoxyethane, Butylisopropylamine, Decyl acetate, and Hexylamine, there is a significant positive correlation between *Bacteroidetes* and Xi-1-Butoxy-1-methoxyethane, Butylisopropylamine, and Decyl acetate (*p* < 0.05). *Synergistetes* are negatively correlated with most of the differential metabolites, among which there is a significant negative correlation with Decyl acetate, Hexylamine, and DG (18:1(11Z)/18:1(9Z)/0:0; *p* < 0.05). At the genus level, *Prevotella* showed a significant negative correlation with most metabolites except Butylisopropylamine, DG (20: 2 (11Z, 14Z)/18: 3 (9Z, 12Z, 15Z)/0: 0), and Decyl acetate, among which DG (20: 2) 11Z Unclassified_o_bacteria showed an extremely significant positive correlation with most of the differential metabolites, including Artemin, Walleminone, Thromboxane B2, and Costunolide.

## 4. Discussion

When ruminants on Qinghai-Tibet Plateau are supplemented during the dry season, the proper nutrition level in the supplementary diet can not only significantly improve the rumen microbial community and metabolites composition of ruminants but also regulate the rumen fermentation index of ruminants, which is the key to improve the growth performance of the host (Yáñez-Ruiz et al., 2015; Xu et al., 2017; Newbold and Ramos-Morales, 2020). Therefore, metagenomics and metabonomics were used to study the effects of different protein levels on the rumen microbial community and metabolites of JY, and the effects on rumen fermentation parameters and growth performance were also discussed.

In recent years, researches on the effect of dietary nutrition level on the growth performance of ruminants showed that the type and nutrition level of diet will have a significant impact on the daily gain and weight of ruminants (Xu et al., 2017; Estrada-Angulo et al., 2018; Wang et al., 2022b). Protein is one of the most important nutrients in ruminants’ diets. Ruminants need to get protein from various feeds in order to maintain their lives and engage in various production. Therefore, the differential supply of protein in supplementary diets may lead to the different growth performances of JY. In this study, the weight of JYs was significantly increased by supplementing diets with different crude protein levels, and the weight of the LP group and HP group was significantly higher than that of the control group. In addition, the weight of the control group showed an obvious downward trend in the last month of the formal experiment, indicating that with the increase in the age of JY, the food intake increased, and the nutrients obtained from pasture during grazing could not meet its growth and development needs, which led to the decline of the weight of JY. Consistent with previous research results, the increase of crude protein levels in the diet can increase the body weight and daily gain of animals (Estrada-Angulo et al., 2018; Qiu et al., 2018; Wang et al., 2020b). Moreover, when feeding lambs with different crude protein levels, the results showed that the daily gain and feed conversion efficiency of lambs were significantly improved by using high protein levels (Karlsson and Martinsson, 2011). In this experiment, the body weight and daily gain of JYs in the HP group were higher than those in the LP group. Therefore, according to the results of this experiment and previous research report (Bihuniak and Insogna, 2015), we speculate that increasing the crude protein level in the diet within a reasonable range can improve the growth performance of animals.

Rumen is a unique structure of ruminants, and it is a living fermentor for ruminants. The stable rumen environment is the basic guarantee for ruminants to digest feed, while pH and VFAs are the main indicators to measure it, and VFAs are the final products of ruminants’ degradation and digestion of protein in diet (Weller et al., 1969; Wang et al., 2012; Melo et al., 2013a). Therefore, in this study, the indexes of VFAs and pH in the rumen were measured. Rumen pH represents the buffering capacity of the rumen, which is used to reflect the stability of the internal environment in the rumen. It is an important index to evaluate rumen fermentation. It is in a dynamic change, with the normal range of 5.5 ~ 7.5, at this time, protozoa and cellulolytic bacteria in the rumen grow rapidly and produce a large number of VFAs to meet the energy needed by animals (Beijer, 1952; Palmonari et al., 2010; Melo et al., 2013a; Mizrahi et al., 2021). In this experiment, the rumen pH in the control group was lower than that in the LP group and the HP group, indicating that the supplementary feeding diet had a certain impact on the rumen internal environment, but the pH among the three groups were within the normal range, indicating that the body itself had certain self-regulation ability. VFAs are the final products of rumen microbials in ruminants when they ferment and degrade diets, and are the main sources of energy metabolism for ruminants’ growth and development (Warner, 1964; Harirchi et al., 2022; Weimer, 2022). In this experiment, the concentrations of Acetate, Propionate, *Iso*-Butyrate, Butyrate, *Iso*-Valerate, and valerate are the lowest in the Control group, which is consistent with other research results (Karimi et al., 2021; Mirzakhani et al., 2021; Yi et al., 2022a). The crude protein level in the supplementary diet has a significant impact on Valerate in JY. The concentration in the LP is higher than that in the HP, which may be related to the internal environment in the rumen of JY. According to previous studies, either too high or too low pH in the rumen can affect rumen absorption of VFAs in ruminants (Calsamiglia et al., 2008). Therefore, we speculate that the difference in acetate concentration among the three groups may be partly related to rumen pH, and the pH among the three groups is different. The ratio of Acetate/Propionate can affect microbial diversity. The ratio of Acetate/Propionate in the rumen is different with different feed types or microbial community structures. The higher the proportion of concentrate in the ruminant diet, the lower the ratio of Acetate/Propionate (Pang et al., 2022a; Yi et al., 2022a). In this experiment, the ratio of Acetate/Propionate in the control group was higher than that in the LP and the HP group, which was consistent with the results of Dai et al. (2022). Therefore, we speculated that the ratio of concentrate in the diet was changed when JYs were supplemented with different crude protein levels, thus reducing the ratio of Acetate/Propionate in the HP and the LP group.

Genomics can study the composition and functional characteristics of rumen microbials, by annotating the genes encoding CAZymes, we can quickly and comprehensively understand the effects of different protein levels of supplementary diets on rumen microflora in JY (Ghurye et al., 2016; Ngara and Zhang, 2018; Taş et al., 2021). In this study, as in previous studies, the dominant microbial compositions of the three treatment groups at phylum level are *Bacteroides*, *Sclerotinia*, *Kiritimatiellaeota*, and *Proteus*. *Bacteroidetes* and *Firmicutes* contain a large number of fiber-degrading bacteria, which can degrade cellulose in feed to produce VFAs, and play an important role in carbohydrate degradation in rumen, thus promoting the growth and development of ruminants (Chen et al., 2022a; Xue et al., 2022b). At the genus level, the dominant microbial genera in the three treatment groups are *Prevotella* and *Ruminococcus*, etc. *Prevotella* can make use of starch and plant cell walls in rumen, such as xylan and pectin. *Ruminococcus* can produce a large amount of cellulase and hemicellulase. More importantly, *Ruminococcus* is an important process involved in the degradation of oligopeptides into amino acids, which is regarded as a restrictive step in protein hydrolysis in rumen (Calsamiglia et al., 2010; Kim et al., 2017; Gaffney et al., 2021; Sharma et al., 2022). There was no significant difference in the relative abundance of these dominant microbials at the phylum and genus levels among the three treatment groups, which may be due to their adaptation to different supplementary diets through the ruminant rumen self-regulation system. It is worth noting that although the relative abundance of *Streptomyces*, *Butyricimonas*, and *unclassified_c_Alphaproteobacteria* is relatively low, these microbials have significant differences between the three treatment groups, which may be due to these differences. The microbial community leads to different degradation capabilities of the supplementary diet between the three treatment groups.

Metagenomics can be used to annotate the genes encoding CAZymes (Kunath et al., 2017); therefore, in this study, the genes encoding CAZymes in rumen microbials were annotated, and it was found that the three treatment groups were GHs and GTs with the largest number. Carbohydrate in the ruminant diet provides fermentation substrate for rumen microorganisms, which can account for more than 60% of the diet. Complex carbohydrates are decomposed into monosaccharides and oligosaccharides under the catalysis of various hydrolases, and then these oligosaccharides are quickly decomposed into pyruvate by microorganisms, which is fermented through metabolic pathways and finally produce acetic acid and propionic acid. Among them, GHs can destroy the glycosidic bond of carbohydrates through hydrolysis, and GTs can catalyze the formation of glycosidic bonds by using sugar donors containing nucleoside phosphate or lipid phosphate leaving groups (Bourne and Henrissat, 2001; Lairson et al., 2008; Ardèvol and Rovira, 2015). CE mainly removes the ester group of polysaccharides and participates in the degradation of the carbohydrate side chain, which can promote GHs and PLs to break the glycosidic bond of polysaccharides (Biely, 2012). PLs can break the glycosidic bond through the mechanism in the body, and the CBMs itself has no catalytic activity, but it can assist GHs and PLs by fixing CAZymes on the substrate surface to increase the contact time and area (Cantarel et al., 2009; Lombard et al., 2010; Jones et al., 2018). There are significant differences among GT28, GT13, GH149, and GH103 among the three treatment groups. In the comparison of CAZymes between LP and HP groups, the number of GH31, GH95, GH67, and GH98 in LP group is significantly higher than that in HP group. Previous studies have shown that GH149 and GH98 play an important role in the degradation of complex carbohydrates (Rigden, 2005; Kuhaudomlarp et al., 2018). Therefore, we speculate that the differences between these CAZymes may affect the degradation process of carbohydrates in the diet of JY, resulting in the difference in valerate concentration in the three groups. Valic acid is the substrate of rumen fermentation, which can be further processed into ruminants as a carbon source, moreover, valeric acid also participates in cellular immunity and is an effective immunomodulator (Nadeau et al., 2003; Feng et al., 2022). Therefore, the difference in valeric acid concentration in the rumen will affect the nutrient absorption capacity of ruminants, which will lead to the difference in growth performance, but the specific mechanism between them needs further study.

In order to further explore the potential relationship between rumen dominant microflora and rumen fermentation parameters and metabolites, Spearman correlation analysis was conducted based on Bray_Curtis distance. According to previous studies, more and more evidences showed that the concentration of metabolites will affect the function of rumen microflora (Zhang et al., 2017; Xue et al., 2020). The results of this experiment are consistent with those of yak and Holstein cattle, indicating that the changes between microbial flora and metabolites are closely related to the health and growth of the body (Zhang et al., 2017, 2021). In this experiment, the results showed that there is a significant positive or negative correlation between dominant microorganisms and differential metabolites. The metabolic pathways of these differential metabolites annotated by the KEGG database mainly include sphingomyelin metabolism, linoleic acid metabolism, histidine metabolism, and bile secretion et al. Deoxycholic acid is a differential metabolite among the three groups, which is significantly related to Proteobacteria. Deoxycholic acid is a steroid and one of the main components of circulating bile acid, which plays an important role in bile secretion (Funabashi et al., 2020). 4-Pentenoic acid is a differential metabolite among the three groups, and it is significantly related to the dominant microorganisms at the phylum level. The metabolite is a lipid and lipid molecule, which has been proved that inhibiting the combination of alanine and glucose in pyruvate carboxylase and glyceraldehyde 3-phosphate dehydrogenase can inhibit the oxidation reaction of fatty acids, thus affecting the growth and development of animals (Gorin and Zendowski, 1975; Zhong et al., 1985). Therefore, we speculate that the dominant microorganisms in JY can change the metabolic pathway in the body by regulating the concentration of these metabolites, and then affect the growth and development of the host.

## 5. Conclusion

In this study, the effects of crude protein level in supplementary diet on growth performance and rumen fermentation parameters of JYs were compared, and the effects on microbial community composition and metabolites were explored by metagenomics and metabolomics. The crude protein level in the supplementary diet significantly increased the daily gain of JY. The pH and concentration of valerate in the LP and HP group were significantly higher than those in the Control group, and the A:P ratio in the Control group was significantly higher than that in the other two groups. The crude protein level in the supplementary diet has no significant effect on the dominant microbial at the taxonomic level of phylum and genus. However, the dominant microorganisms are closely related to the different metabolites in serum and rumen, which are related to bile secretion and histidine metabolism, and are closely related to rumen fermentation parameters. To sum up, it is necessary to supplement the diet with suitable crude protein levels for JY during the dry season. This study is helpful to further explore the microbial and metabolic functions of JY fed with different crude protein levels, and then understand the protein requirements of JY in the dry season, so as to provide basic data for formulating more scientific supplementary diets in the future.

## Data availability statement

The datasets presented in this study can be found in online repositories. The names of the repository/repositories and accession number(s) can be found at: NCBI: PRJNA904725.

## Ethics statement

The animal study was reviewed and approved by Lanzhou Institute of Husbandry and Pharmaceutical Sciences of the Chinese Academy of Agricultural Sciences. Written informed consent was obtained from the owners for the participation of their animals in this study.

## Author contributions

CL, PY, and RFD contributed to conception and design of this study. RFD, RQD, XYM, and CH conducted relevant experiments. RFD drafted the manuscript. RFD, XYM, XMM, and YL organized the database. RFD and CH participated in the editing of the manuscript. CL and PY provided the funding supports. All authors contributed to the article and approved the final manuscript.

## Funding

This work was supported by the State Key R&D program (2021YFD1600200); Gansu basic research innovation group project (20JR5RA580); Major science and technology projects in Gansu Province (21ZD10NA001, GZGG-2021-1); Modern beef yak industry technology system (MATS-Beef Cattle System, CARS-37); and Yak resources and breeding innovation project of Chinese Academy of Agricultural Sciences (25-LIHPS-01).

## Conflict of interest

The authors declare that the research was conducted in the absence of any commercial or financial relationships that could be construed as a potential conflict of interest.

## Publisher’s note

All claims expressed in this article are solely those of the authors and do not necessarily represent those of their affiliated organizations, or those of the publisher, the editors and the reviewers. Any product that may be evaluated in this article, or claim that may be made by its manufacturer, is not guaranteed or endorsed by the publisher.

## Footnotes

^1^ http://www.hmdb.ca./
