# Supplementary material for: Effects of dietary crude protein levels in the concentrate supplement after grazing on rumen microbiota and metabolites by using metagenomics and metabolomics in Jersey-yak
Source: Front Microbiol. 2023 May 2;14:1124917. doi: 10.3389/fmicb.2023.1124917 (PMC10185794; doi:10.3389/fmicb.2023.1124917)

Supplementary Material

# Supplementary Material S1

Supplementary Material S1 Composition and content of supplementary diet

| Raw material name | The content of treatment/% | |
| --- | --- | --- |
|  | LP | HP |
| Corn | 50.2 | 53 |
| Barley | 15.5 | 11.6 |
| Palm meal | 10.5 | 10 |
| Molasses | 3 | 0.7 |
| Large pieces of soybean meal | 7.9 | 7.7 |
| Cottonseed meal | 8 | 11.6 |
| Expanded urea | 0.7 | 1.2 |
| Feed grade magnesium oxide | 0.2 | 0.2 |
| Calcium hydrogen phosphate | 1.5 | 1.5 |
| Sodium bicarbonate | 0.6 | 0.6 |
| Feed grade sodium chloride | 1 | 1 |
| Fine stone powder | 0.8 | 0.8 |
| Ruminant premix feed | 0.1 | 0.1 |
| Nutrient levels | | |
| Crude protein | 15.16 | 17.90 |
| Ether extract | 3.69 | 3.99 |
| Ca | 0.85 | 0.84 |
| P | 0.66 | 0.66 |
| Metabolizable energy | 10.12 | 10.19 |
| Net energy for weight gain | 4.37 | 4.41 |

# Supplementary Material S2

Supplementary Material S2: Comparison of body weight and monthly weight change among three treatment groups

| Item | Treatment group (unit: kg, Mean±SEM） | | | *P*-Value |
| --- | --- | --- | --- | --- |
|  | Control | LP | HP |  |
| Month 0 | 62.23±5.35^b^ | 65.61±4.36^ab^ | 68.50±4.32^a^ | 0.0203 |
| Month 1 | 65.57±4.58^c^ | 73.37±4.05^b^ | 80.33±4.57^a^ | 0.0022 |
| Month 2 | 69.20±4.60^b^ | 81.23±4.04^a^ | 86.20±3.84^a^ | 0.0030 |
| Month 3 | 71.98±4.96^b^ | 86.25±4.51^a^ | 91.08±3.89^a^ | 0.0133 |
| Month 4 | 70.46±5.07^b^ | 89.03±5.10^a^ | 95.00±3.13^a^ | 0.0064 |
| ∆ Month 1 | 3.43±2.08^b^ | 7.75±1.51^ab^ | 11.83±1.30^a^ | 0.0144 |
| ∆ Month 2 | 3.53±1.66 | 7.87±1.37 | 5.87±1.26 | 0.1653 |
| ∆ Month 3 | 2.78±1.09 | 5.02±1.27 | 4.88±1.32 | 0.4592 |
| ∆ Month 4 | -1.52±0.45^b^ | 2.78±1.69^ab^ | 3.92±1.66^a^ | 0.0703 |
| Average daily gain | 0.069±0.038^b^ | 0.195±0.042^a^ | 0.22±0.025^a^ | 0.0297 |

# Supplementary Material S3

Supplementary Material S3: Statistical analysis of metagenomic sequencing data of each sample

| Treatment group | Sample name | Raw reads | Clean reads | Contigs | N50 (bp) | ORFs |
| --- | --- | --- | --- | --- | --- | --- |
| Control | Control 1 | 48,120,162 | 47,274,246.00 | 593,706.00 | 798.00 | 794,190.00 |
|  | Control 2 | 45,519,984 | 44,791,220.00 | 539,069.00 | 792.00 | 733,409.00 |
|  | Control 3 | 51,500,858 | 50,830,440.00 | 706,626.00 | 774.00 | 943,286.00 |
|  | Control 4 | 47,670,398 | 47,005,938.00 | 713,341.00 | 685.00 | 914,343.00 |
|  | Control 5 | 49,437,296 | 48,773,824.00 | 713,951.00 | 738.00 | 937,821.00 |
|  | Control 6 | 45,109,946 | 44,360,594.00 | 698,429.00 | 787.00 | 907,189.00 |
| LP | LP 1 | 45,905,498 | 45,327,352.00 | 680,448.00 | 715.00 | 873,696.00 |
|  | LP 2 | 42,804,918 | 42,030,624.00 | 683,199.00 | 694.00 | 862,977.00 |
|  | LP 3 | 49,349,312 | 48,447,866.00 | 695,421.00 | 730.00 | 901,206.00 |
|  | LP 4 | 42,514,444 | 41,928,174.00 | 499,540.00 | 747.00 | 660,789.00 |
|  | LP 5 | 58,851,406 | 57,885,664.00 | 566,689.00 | 1,059.00 | 821,742.00 |
|  | LP 6 | 46,417,486 | 45,555,776.00 | 605,717.00 | 745.00 | 799,805.00 |
| HP | HP 1 | 47,557,492 | 46,663,136.00 | 595,617.00 | 939.00 | 828,093.00 |
|  | HP 2 | 51,618,068 | 50,799,456.00 | 637,246.00 | 835.00 | 862,681.00 |
|  | HP 3 | 42,849,110 | 42,061,330.00 | 706,082.00 | 623.00 | 860,717.00 |
|  | HP 4 | 56,279,984 | 55,520,902.00 | 849,226.00 | 702.00 | 1,103,962.00 |
|  | HP 5 | 48,996,968 | 48,349,102.00 | 694,854.00 | 720.00 | 906,261.00 |
|  | HP 6 | 44,170,604 | 43,633,690.00 | 621,644.00 | 813.00 | 836,831.00 |
| Total | | 864,673,934 | 851,239,334.00 | 11,800,805.00 | 13,896.00 | 15,548,998.00 |
| Mean | | 48,037,441 | 47,291,074.11 | 655,600.28 | 772.00 | 863,833.22 |
| SD | | 4,449,071 | 4,397,910.26 | 81,518.10 | 99.47 | 93,352.90 |

# Supplementary Material S4

Supplementary Material S4: Microbial composition of all samples at domain taxonomic level


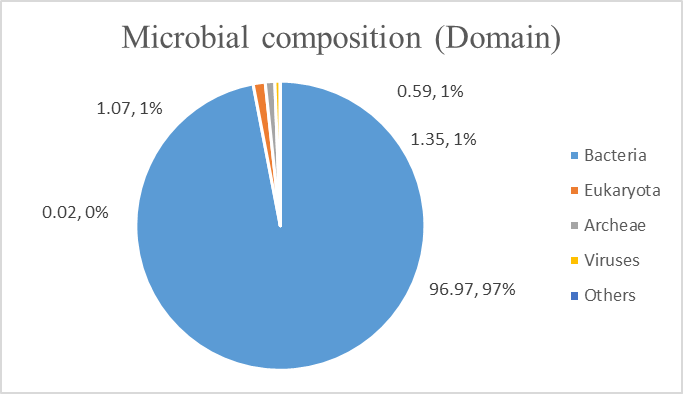

Supplement: Supplementary file 1 [file Table_1.DOCX]
